# Supplementary material for: Willingness to use electronic medical record (EMR) system and its associated factors among health professionals working in Amhara region Private Hospitals 2021, Ethiopia
Source: PLoS One. 2023 May 1;18(5):e0282044. doi: 10.1371/journal.pone.0282044 (PMC10150984; doi:10.1371/journal.pone.0282044)
Supplement: S1 Questionnaire — (DOCX) [file pone.0282044.s002.docx]

**Informed Consent of Statement**

Hello! My name is __________________________I am here on behalf of ANDUALEM FENTAHUN, student of Master of Public Health at the University of Gondar College of Medicine and Health Sciences, Institute of Public Health. He is conducting research for partial fulfillment of a master degree on **Willingness To Use Electronic Medical Record (EMR) System And Its Associated Factors Among Health Professionals Working In Amhara Region Private Hospitals**, 2020\21. He has ethical clearance from the University of Gondar. You are chosen to participate in this study and your anonymous answers will be used only for research purposes. In order to effectively attain the purpose of the research, I request you to give a genuine response to each question. There are questions for you to complete and there is no need to put your name on the questionnaire; no individual responses will be reported. Your answers are completely confidential. It is your full right to refuse, to answer any or all of the questions. If you don't want to participate you can leave the questionnaire empty. Study questionnaires will take a maximum of 15 minutes.

**Certificate of consent:** I understand that the findings of this research will be disseminated to private Hospital management and decision-makers that will be useful as an input for the next actual use of EMRs among health professionals and sustainability utilization of EMRs.

I voluntarily consent to participate in this study.

I agree I disagree

If you are agree to participate in the study please visit the next page.

For any further question, contact the investigator

Name of the investigator: ANDUALEM FENTAHUN, Phone Number: 0923551141

Thank you for consenting to be a participant in this study

**For Data collectors use only**

Facility name ________________________ Questionnaire ID: ___________

Data collector name_________________________ date____________ signature__________

**Part 1. Socio- Demographic Characteristics of the Study Subject**

| No | Questions | Responses | | Skip to |
| --- | --- | --- | --- | --- |
| 101 | Your sex. | 1. Male 2. Female | |  |
| 102 | Your age in years | …………….. | |  |
| 103 | Your religion | 1.Orthodox Christian 2. Muslim  3. Others | |  |
| 104 | Marital status | 1.Single 2. Married 3. Divorce 4. Widow | |  |
| 105 | Educational level | 1. Diploma 2. Degree.  3. Master 4. Specialist and above | |  |
| 106 | Your professional category. | 1. Physician 2. Nurse  3. Laboratory 4. Pharmacy  5. Midwifery 6. Others. | |  |
| 107 | Working ward/unit | ……………………… | |  |
| 108 | Monthly income in Ethiopia birr | ……………………… | |  |
| 109 | Total Work experience in years (if <1year in month) | ……………………… | |  |
| 110 | Have you experiencing language difficulties when using computer system (EMR)? | 1. Yes 2. No | |  |
| 111 | Have you received any form of training course on EMR software? | 1. Yes 2. No | | If yes skip 112 |
| 112 | If No, for que111, what is your main reason not taking the training? | 1.I don’t have the time to take training  2. I have no access to take training  3. I’m not interested to take training  4. My work does not need training " | |  |
| 113 | Do you have full computer (laptop or desktop) access? | 1. Yes 2. No | | If no skip 114 |
| 114 | If your answer is “yes” for que 113, where is your computer access? | 1. At home 2. At work place 3. Both | |  |
| 115 | If your answer is “yes” for que 113 Do you have full printer access in your work area? | 1. Yes 2. No | |  |
| 116 | Have you use a computer in your work place? | 1. Yes 2. No | | If no skip 117-119 |
| 117 | If your answer is “yes” for que116, how often do you use computer? | In every healthcare delivery  1. Daily 2. Weekly 3. Three times a week 4. Monthly 5. Not specific. | |  |
| 118 | If “yes” for que 116, How long have you been using the computer? | ……………………….. | |  |
| 119 | If your answer is “yes” que 116, mainly for what purpose do you use computer? | 1. Report writing 4. Keeping patient file  2. Listening music 5. Using internet  3. Reading 6. Others | |  |
| 120 | Do you have local area internet network access for using EMR? | 1. Yes 2. No | | If no skip 121-125 |
| 121 | If your answer is “yesque 120, Are your department computers linked local area internet for using EMR? | 1. Yes 2. No | |  |
| 122 | If your answer is “yes” que 121, Do you use local area internet for EMR? | 1. Yes 2. No | |  |
| 123 | If your answer is “yes” que 122, how often do you use internet service? | 1. Every movement of patient data mgt  2. Every data  3. At least once a week  4. At least once a month | |  |
| 124 | If your answer is “yes” que 122, mainly for what purpose do use internet? | 1. To get information which support my profession.  2. To communicate with my friends  3. To get daily news  4. To manage patient’s health profile and data  5. To report different data  6. Other………… | |  |
| 125 | Does your hospital have trained IT technical support personnel? | | 1. Yes 2. No | If no skip 126 |
| 126 | If your answers yes que 125, is there Provision of technical support? | | 1. Yes 2. No |  |
| 127 | Do you think that EMR system reduce patient waiting time? | | 1. Yes 2. No |  |
| 128 | Do you have EMR implementation guideline in your health institution? | | 1. Yes 2. No |  |
| 129 | Do you have budget allocation to implement EMR? | | 1. Yes 2. No |  |
| 130 | Is there supportive supervision (M&E) for effective EMR implementation? | | 1. Yes 2. No |  |
| 131 | Is there management support to implement EMR of new technology? | | 1. Yes 2. No |  |

**Part 2. Health professional Willingness to Use EMR**

| No | Statements | Response | Skip |
| --- | --- | --- | --- |
| 201 | Do you have willingness to undergo computer training to enable your usage of EMRs? | 1. Yes 2. No |  |
| 202 | Do you have willingness to purchase a personal laptop/computer and use to familiarize yourself with the usage of EMR? | 1. Yes 2. No |  |
| 203 | Do you have willingness to undergo trainings on EMRs & its implementation? | 1. Yes 2. No |  |
| 204 | Do you have willingness to use EMRs if properly trained? | 1. Yes 2. No |  |
| 205 | Do you have willingness to use EMRs if the technical infrastructures are available? | 1. Yes 2. No |  |

**Part 3. Computer skill**

For part 3 and 4 please point out the extent about EMR, which you agree with each of the following statements. Please tick (√) or circle the appropriate answer.

Use liker scale with strongly disagree- 1, disagree- 2, Neutral -3, Agree-4 and strongly agree-5

| No | Factor | Strongly disagree | Disagree | Neutral | Agree | Strongly agree |
| --- | --- | --- | --- | --- | --- | --- |
| 301 | I am competent in using MS Window | 1 | 2 | 3 | 4 | 5 |
| 302 | I am competent in using MS Word | 1 | 2 | 3 | 4 | 5 |
| 503 | I am competent in using MS Excel | 1 | 2 | 3 | 4 | 5 |
| 304 | I am competent in using MS Access | 1 | 2 | 3 | 4 | 5 |
| 305 | I am competent in using power point | 1 | 2 | 3 | 4 | 5 |
| 306 | I am competent in using access and browse internet. | 1 | 2 | 3 | 4 | 5 |

**Part 4. Knowledge about to use EMR**

| No | Questions | Strongly disagree | Disagree | Neutral | Agree | Strongly agree |
| --- | --- | --- | --- | --- | --- | --- |
| 401 | I have good proficiency in computer use | 1 | 2 | 3 | 4 | 5 |
| 402 | I am familiar with the concept of EMR | 1 | 2 | 3 | 4 | 5 |
| 403 | I am familiar to the benefits of EMR | 1 | 2 | 3 | 4 | 5 |
| 404 | I am familiar with tools and technologies needed for EMR | 1 | 2 | 3 | 4 | 5 |
| 405 | I am familiar with EMR of other countries | 1 | 2 | 3 | 4 | 5 |
| 406 | Education has effect in increasing the knowledge of hospital staff to work with EMR. | 1 | 2 | 3 | 4 | 5 |
| 407 | EMR has effect in increasing the quality of health care services and patient safety | 1 | 2 | 3 | 4 | 5 |
| 408 | EMR has effect in increasing the speed to deal with patients. | 1 | 2 | 3 | 4 | 5 |
| 409 | EMR has effect in reducing the patients and hospital costs | 1 | 2 | 3 | 4 | 5 |
| 410 | EMR has effect on public access to healthcare services | 1 | 2 | 3 | 4 | 5 |
| 411 | EMR has effect in increasing patients’ satisfaction | 1 | 2 | 3 | 4 | 5 |
| 412 | EMR has effect in preserving the patients’ privacy | 1 | 2 | 3 | 4 | 5 |
| 413 | EMR has effect in preserving the confidentiality and security of patient information. | 1 | 2 | 3 | 4 | 5 |
