# Supplementary material for: Willingness to use electronic medical record (EMR) system and its associated factors among health professionals working in Amhara region Private Hospitals 2021, Ethiopia
Source: PLoS One. 2023 May 1;18(5):e0282044. doi: 10.1371/journal.pone.0282044 (PMC10150984; doi:10.1371/journal.pone.0282044)

PLOS ONE

Willingness to Use Electronic Medical Record (EMR) System and its Associated Factors among Health Professionals Working in Amhara region Private Hospitals 2021, Ethiopia

--Manuscript Draft--

**Manuscript Number:**	PONE-D-22-11490
**Article Type:**	Research Article
**Full Title:**	Willingness to Use Electronic Medical Record (EMR) System and its Associated Factors among Health Professionals Working in Amhara region Private Hospitals 2021, Ethiopia
**Short Title:**	Willingness to Use Electronic Medical Record System and its Associated Factors among Health Professionals
**Corresponding Author:**	Andualem Fentahun Senishaw, MPH<br>Debre Markos University<br>Debre Markos, ETHIOPIA
**Keywords:**	Willingness; Private; Health Professionals; Electronic Medical Record System
**Abstract:**	Introduction<br>Despite the high expectation in EMR as a great prospect for improving performance in healthcare, the level of adoption and utilization particularly in a developing country is low. Knowing the willingness to use the EMR system in the private hospital has an impact on the future implementation status and utilization of the EMR in Ethiopia. However, there was no evidence of the status on willingness to use electronic medical record system in private hospitals in the Amhara region. This study aimed to assess the willingness to use electronic medical record Systems and its associated factors among health professionals working in Amhara Region Private Hospitals.<br>Methods<br>A cross-sectional institutional study was performed among 406 health professionals selected using proportional allocation with simple random sampling technique in Amhara region private hospitals by using self-administered structured questionnaires. The data were analyzed using SPSS version 20 software. Descriptive statistics with Binary logistic regression was performed to estimate the crude and adjusted odds ratio with a 95% Confidence interval.<br>Results<br>Out of 406 participants included in the analysis, 307 (75.6%) participants showed a willingness to use the EMR system. 312 (76.8%) health professionals had good knowledge of EMR systems and 257 (63.3%) had good computer skills on EMR systems. health professionals who had EMR Knowledge (AOR=1.85, 95% CI (1.004-3.409)), EMR Training (3.29, 95% CI (1.353-8.003)), technical support personnel (1.92, 95% CI (1.122-3.305)), supportive supervision (AOR=1.97, 95% CI (1.072-3.628)), and Computer skill on EMR (1.77, 95% CI (1.002-3.148)) were significantly associated with the outcome variable.<br>Conclusions<br>This finding demonstrates a high proportion of willingness to use the EMR system. Knowledge, skill, training, technical support personnel, and supportive supervision were significantly associated with the outcome variable. Stockholders should focus on continuous follow-up, adequate training, and technical support of health professionals.
**Order of Authors:**	Andualem Fentahun Senishaw, MPH
**Additional Information:**	
**Question**	**Response**
**Financial Disclosure**<br><br>Enter a financial disclosure statement that describes the sources of funding for the work included in this submission. Review	The author(s) received no specific funding for this work.

the submission guidelines for detailed requirements. View published research articles from *PLOS ONE* for specific examples.<br><br>This statement is required for submission and **will appear in the published article** if the submission is accepted. Please make sure it is accurate.<br><br>**Unfunded studies**<br>Enter: *The author(s) received no specific funding for this work.*<br><br>**Funded studies**<br>Enter a statement with the following details:<br>• Initials of the authors who received each award<br>• Grant numbers awarded to each author<br>• The full name of each funder<br>• URL of each funder website<br>• Did the sponsors or funders play any role in the study design, data collection and analysis, decision to publish, or preparation of the manuscript?<br>• **NO** - Include this sentence at the end of your statement: *The funders had no role in study design, data collection and analysis, decision to publish, or preparation of the manuscript.*<br>• **YES** - Specify the role(s) played.<br><br>* typeset	
**Competing Interests**<br><br>Use the instructions below to enter a competing interest statement for this submission. On behalf of all authors, disclose any competing interests that could be perceived to bias this work—acknowledging all financial support and any other relevant financial or non-financial competing interests.<br><br>This statement is required for submission and **will appear in the published article** if the submission is accepted. Please make sure it is accurate and that any funding sources listed in your Funding Information later in the submission form are also	The authors have declared that no competing interests exist.

declared in your Financial Disclosure statement.<br><br>View published research articles from *PLOS ONE* for specific examples.<br><br>**NO authors have competing interests**<br><br>Enter: *The authors have declared that no competing interests exist.*<br><br>**Authors with competing interests**<br><br>Enter competing interest details beginning with this statement:<br><br>*I have read the journal's policy and the authors of this manuscript have the following competing interests: [insert competing interests here]*<br><br>* typeset	
**Ethics Statement**<br><br>Enter an ethics statement for this submission. This statement is required if the study involved:<br><br>• Human participants<br>• Human specimens or tissue<br>• Vertebrate animals or cephalopods<br>• Vertebrate embryos or tissues<br>• Field research<br><br>Write "N/A" if the submission does not require an ethics statement.<br><br>General guidance is provided below. Consult the submission guidelines for detailed instructions. **Make sure that all information entered here is included in the Methods section of the manuscript.**	Ethical clearance was obtained from the ethical review committee of the University of Gondar College of Medicine and Health Science, Institute of Public Health by REF NO IPH 1319/2021.<br>Informed written consent was obtained from each study participant after telling the objective of the study. They were also informed about the benefits of the study. The data collection was anonymous and the information was kept confidential.

Powered by Editorial Manager® and ProduXion Manager® from Aries Systems Corporation

**Format for specific study types**<br><br>**Human Subject Research (involving human participants and/or tissue)**<br>• Give the name of the institutional review board or ethics committee that approved the study<br>• Include the approval number and/or a statement indicating approval of this research<br>• Indicate the form of consent obtained (written/oral) or the reason that consent was not obtained (e.g. the data were analyzed anonymously)<br><br>**Animal Research (involving vertebrate animals, embryos or tissues)**<br>• Provide the name of the Institutional Animal Care and Use Committee (IACUC) or other relevant ethics board that reviewed the study protocol, and indicate whether they approved this research or granted a formal waiver of ethical approval<br>• Include an approval number if one was obtained<br>• If the study involved *non-human primates*, add *additional details* about animal welfare and steps taken to ameliorate suffering<br>• If anesthesia, euthanasia, or any kind of animal sacrifice is part of the study, include briefly which substances and/or methods were applied<br><br>**Field Research**<br><br>Include the following details if this study involves the collection of plant, animal, or other materials from a natural setting:<br>• Field permit number<br>• Name of the institution or relevant body that granted permission	
**Data Availability**<br><br>Authors are required to make all data underlying the findings described fully available, without restriction, and from the time of publication. PLOS allows rare exceptions to address legal and ethical concerns. See the PLOS Data Policy and FAQ for detailed information.	Yes - all data are fully available without restriction

A Data Availability Statement describing where the data can be found is required at submission. Your answers to this question constitute the Data Availability Statement and **will be published in the article**, if accepted.<br><br>**Important:** Stating 'data available on request from the author' is not sufficient. If your data are only available upon request, select 'No' for the first question and explain your exceptional situation in the text box.<br><br>Do the authors confirm that all data underlying the findings described in their manuscript are fully available without restriction?	
**Describe where the data may be found in full sentences. If you are copying our sample text, replace any instances of XXX with the appropriate details.**<br><br>• If the data are **held or will be held in a public repository**, include URLs, accession numbers or DOIs. If this information will only be available after acceptance, indicate this by ticking the box below. For example: *All XXX files are available from the XXX database (accession number(s) XXX, XXX.).*<br>• If the data are all contained **within the manuscript and/or Supporting Information files**, enter the following: *All relevant data are within the manuscript and its Supporting Information files.*<br>• If neither of these applies but you are able to provide **details of access elsewhere**, with or without limitations, please do so. For example:<br><br>*Data cannot be shared publicly because of [XXX]. Data are available from the XXX Institutional Data Access / Ethics Committee (contact via XXX) for researchers who meet the criteria for access to confidential data.*<br><br>*The data underlying the results presented in the study are available from (include the name of the third party*	relevant data are within the manuscript and its Supporting Information files.

and contact information or URL).<br>• This text is appropriate if the data are owned by a third party and authors do not have permission to share the data.<br><br>* typeset	
Additional data availability information:	

Powered by Editorial Manager® and ProduXion Manager® from Aries Systems Corporation

manuscript

Click here to access/download;Manuscript;willingness to use EMR PLOSE ONE.docx

Willingness to Use Electronic Medical Record (EMR) System and its Associated Factors among Health Professionals Working in Amhara region Private Hospitals 2021, Ethiopia

Andualem Fentahun Senishaw[1*], Biniyam ChakiluTilahun[2‡], Araya Mesfin Nigatu[2‡], Shegaw Anagaw Mengiste[3‡,] Karen Standal[3‡]

[1]Department of health informatics, College of Health Sciences, Debre Markos University, Debre Markos, Ethiopia

[2]Department of health informatics, Institute of Public Health, College of Medicine and Health Science, University of Gondar, Gondar, Ethiopia

[3]School of Business, Institute of Business, History and Social Science, University of South-Eastern Norway, Notodden, Norway

* andugissa@gmail.com (AF)

‡ BT, AM, SM and KS are second equal contribution to this work.

Abstract

Introduction

Despite the high expectation in EMR as a great prospect for improving performance in healthcare, the level of adoption and utilization particularly in a developing country is low. Knowing the willingness to use the EMR system in the private hospital has an impact on the future implementation status and utilization of the EMR in Ethiopia. However, there was no evidence of the status on willingness to use electronic medical record system in private hospitals in the Amhara region. This study aimed to assess the willingness to use electronic medical record Systems and its associated factors among health professionals working in Amhara Region Private Hospitals.

Methods

A cross-sectional institutional study was performed among 406 health professionals selected using proportional allocation with simple random sampling technique in Amhara region private hospitals by using self-administered structured questionnaires. The data were analyzed using SPSS version 20 software. Descriptive statistics with Binary logistic regression was performed to estimate the crude and adjusted odds ratio with a 95% Confidence interval.

Results

Out of 406 participants included in the analysis, 307 (75.6%) participants showed a willingness to use the EMR system. 312 (76.8%) health professionals had good knowledge of EMR systems and 257 (63.3%) had good computer skills on EMR systems. health professionals who had EMR Knowledge (AOR=1.85, 95% CI (1.004-3.409)), EMR Training (3.29, 95% CI (1.353-8.003)), technical support personnel (1.92, 95% CI (1.122-3.305)), supportive supervision (AOR=1.97, 95% CI (1.072-3.628)), and Computer skill on EMR (1.77, 95% CI (1.002-3.148)) were significantly associated with the outcome variable.

Conclusions

This finding demonstrates a high proportion of willingness to use the EMR system. Knowledge, skill, training, technical support personnel, and supportive supervision were significantly associated with the outcome variable. Stockholders should focus on continuous follow-up, adequate training, and technical support of health professionals.

Introduction

All over the world, Health Information Systems (HIS) and technologies are getting used increasingly and are seen as a good way to increase health system performance and the quality of patient care(1). HIS enable healthcare worker to document relevant patient records and offer an efficient and advanced healthcare service(2). Healthcare organizations achieve these outcomes through the use of HIS such as Electronic Medical Records (EMR), telehealth, mobile health applications, and health information management systems (3). The most important contribution of EMR is that a patient can have one electronic data that can be accessed at any time within a single health care delivery organization and also EMR enables faster work, Fewer errors, Prevent the loss of data, Ensuring confidentiality, Continuity of care, and improving the overall health care system but in the same wise there are potential cons such as Security, Confidentiality and others like crashes and power-cuts (4).

The national wide e-health approach toolkit developed through the world health organization (WHO) and International Telecommunication Union (ITU)(5) defines EMR as “a computerized medical record used to capture, store, and share information among healthcare providers in an organization, supporting the delivery of health services to patients”.

even though there may be an excessive expectation and need in EMR as a great prospect for improving high-quality, continuity, safety, and performance in healthcare, the general adoption status particularly in a developing country is low(6). Many reasons can be given to the low utilization and adoption of EMR in

Ethiopia, but the main ones are related to skill gap, Lack of awareness and knowledge, Lack of availability of technical support and infrastructure, perception, willingness, attitude, technology resistance, and administrative problems have slowed the process of implementation and adoption of EMR (7, 8). The unwillingness of health professionals to accept and utilize digital health information systems and electronic medical records might be one of the essential obstacles that hinder the successful implementation and utilization of electronic health information systems.

Knowing the willingness to use the EMR system in private and public hospitals has an impact on the future implementation status and utilization of the EMR system in Ethiopia(9). Particularly this study focuses on the private health facilities since the attention was not given to the private hospitals towards the willingness of EMR system with evidence-based research. The Ethiopian government needs to digitalize public as well as private health facilities and also needs to strengthen the public-private partnership. Public-private partnership in health (PPPH) was an element of the Ethiopian health sector transformation plan (10, 11). The overall policy environment supports private sector engagement and public-private partnerships(12, 13). Health Policies and strategies in Ethiopia are focused on the material aspect particularly the installations and infrastructure of EMR systems and have ignored the attitudinal and behavioral factors (willingness) of healthcare professionals in using the EMR system in private as well as public hospitals(9).

To overcome the issues related to EMR and to facilitate the adoption and use of innovative technologies, it is very important to understand the factors that affect the willingness to use the EMR system by health professionals in healthcare. Health professionals' understanding, knowledge, willingness, and mindset on EMR use are vital elements that could impact its future fulfillment (14).

Studies have shown that a lack of knowledge, skills, availability of skilled human capital, resource availability, computer literacy, English language proficiency,

educational status, and training are the factors that potentially affect the willingness to use EMR systems by the healthcare professionals(9). Most of the problems are related to the unwillingness of its users to accept the transition from paper-based to electronic systems. This has contributed to the failure costs of many EMR installations (6).

In the Ethiopian health system, the majority of private health facilities are found in the urban area and also majority population seeks health service from the private health facility, but most of the time the data documentation process regarding quality is not the same as the government health facilities because the private health facilities are not taking the mandates as the governmental hospital to report the data to the concerned body or FMOH (federal ministry of health). With the burden of increased service users and disease private hospitals' information quality and utilization for decision-making remain vulnerable and weak(9), this is because private EMR infrastructure is stimulated by a reluctance to use already existing technology (15).

Having electronic medical records systems in many health organizations improves the efficiency of health care delivery and availability of health information at any time and also retrieves patient information for patient care, statistics, research, and teaching (16, 17). Therefore adopting EMR in a private health facility is one of the digital technologies that can successfully transform the health system by reducing the public or governmental health facility burden by making the service fast, easy and accurate in the private sector.

There is no study conducted in the Ethiopian context on the willingness to use EMR among health professionals working in private hospitals. Therefore, this study aims to assess the willingness to use electronic medical record (EMR) systems and its associated factors among health professionals working in Amhara Region Private Hospitals.

Materials and Methods

Study design and setting

A cross-sectional institutional study was performed from January 25 to February 20, 2021. The study was conducted in private hospitals found in the Amhara region. The Amhara Region is located in the northwestern part of Ethiopia and the region is divided into 13 zones, and 140 Weredas. This region Has a total of 4490 public health facilities (81 hospitals, 859 health centers, and 3550 health posts) and 1165 private health facilities (1157 clinics, 10 hospitals). The distributions of these 10 hospitals are four from Bahirdar town woreda HO, three from Dessie town woreda HO, two from north Shewa ZHD, and 1 from Gondar town Woreda HO. These private hospitals are Yifat hospital, Afilas hospital, Salam hospital, Adina's hospital, Ayu hospital, Bati hospital, Dream care hospital, Ethio hospital, Gamby hospital, and IBEX hospital.

Sample size determinations, study participants and Sampling procedures

The sample size of this study was determined using a single population proportion formula: considering the following assumptions: 95% level of confidence, 5% margin of error, and 10% non-response rate, proportion (p) of 50% of willing to use the EMR system. Finally, a sample size of 423 was obtained. The source population was all health professionals working in private hospitals in the Amhara region and the study population was selected health professionals who had been working in ten private hospitals of the Amhara region. Health professionals who were working less than six months and who were working both at private and governmental health facilities were excluded to avoid bias.

There were ten private hospitals in the Amhara region. From each hospital permanent health professionals working during the study period were included in the study; proportional allocation was done for each hospital according to the

actual number. Each healthcare worker in each private hospital was selected by simple random sampling from a list of medical staff administrative records.

Operational definition

Knowledge: - Knowledge was measured on a 5-point Likert scale ranging from ‘strongly disagree’ (score 1) to ‘strongly agree’ (score 5). Thirteen knowledge questions were added and divided by 13 to create a composite variable scale (ranging from score 1 to 5) for data analysis. Finally, the composite variable score was dichotomized as ‘good knowledge’ or ‘poor knowledge’ based on the final score. Accordingly, a final score of the above three (agree and strongly agree) were categorized as ‘good knowledge’ while those final scores of three or below (strongly disagree, disagree, and neutral) were categorized as ‘poor knowledge’ (18)

Willingness to use**:-** Willingness of health professionals to use the EMR system was rated into “willing” and “not willing” using composite scores obtained from all the five willingness questions (9), Five questions were scored, and the maximum score obtainable is 5 marks. A score of 3 marks and above out of 5 marks suggests willingness while a score of less than 3 marks suggests an unwillingness to use the EMR system (9).

Computer skill**: -** skill was measured on a 5-point Likert scale ranging from ‘strongly disagree’ (score 1) to ‘strongly agree’ (score 5). Each skill question was added and divided by the number of questions to create a composite variable scale (ranging from score 1 to 5) for data analysis. Accordingly, a final score of above three (agree and strongly agree) was categorized as ‘good skill’ while those final scores of three or below (strongly disagree, disagree, and neutral) were categorized as ‘poor skill’ (18).

Data collection tools and procedures

Self-administered structured questionnaires were used to gather quantitative data by using questionnaires adapted from (9, 19). The outcome variable

includes five questions, respondents were asked to indicate either ‘Yes’ or ‘No’ and some multiple-choice questions. Socio-demographic, technical, organizational, and resource-related variables were included in the questionnaire. A questionnaire was prepared in English this is because the study participants were well educated and they can easily understand English and if the questioner translated into the local language (Amharic) it might create some difficulty to understand. Health information technicians (HIT) who have good communication skills were recruited for collecting questioner. Health officers who were experienced in research work supervised the data collection process. A two-day training was given for facilitators and supervisors on the objective of the study, data collection procedures, data collecting tools, respondents’ approach, data confidentiality, and respondents right before the data collection date. The completeness of the questionnaires and the overall quality of data collected and the daily status of the data collector were checked every day by the supervisors and investigator. Before the actual data collection, pretesting of the questionnaire was conducted outside the study area but with similar characteristics to the actual study area.

Data management, analysis and quality assurance

Data was entered using Epi-data version 4.6 and analyzed using Statistical Package for Social Science (SPSS) version 20. Descriptive analyses were computed for all variables in the study. Bivariable and multivariable analysis with adjusted odds ratio was used to measure the association of dependent and independent variables, 95% confidence intervals, and P-value was calculated to evaluate statistical significance. Variables with a p-value of ≤ 0.2 on binary logistic regression analysis were entered and further computed on the multivariable logistic regression model. Adjusted odds ratios and 95% confidence intervals were calculated for each of the independent variables in logistic regression models with the health professional willingness to use the EMR system as a dependent variable.

Based on the pretest finding internal consistency for each dimension of the composite variable questionnaire was checked using Cronbach's alpha. Score on EMR knowledge (Cronbach alpha=0.79), computer skill (Cronbach alpha=0.91), willingness to use EMR system (Cronbach alpha=0.81). Based on this they were acceptable. The goodness of fit of the model was assessed using Hosmer Lemeshow's statistical test and its values above 5% which is 0.76 indicate that the model has a good predictive ability. A multicollinearity test was performed for the variables included in the final multivariable model. Hence, the variables had a VIF value of less than five.

Ethical approval and consent to participate

The study protocol was reviewed and obtained from the ethical review committee of the University of Gondar College of Medicine and Health Science, Institute of Public Health. A supporting letter was also obtained from the Amhara Regional Health bureau. Informed written consent was obtained from each study participant after telling the objective of the study. They were also informed about the benefits of the study. The data collection was anonymous and the information was kept confidential.

Results

Socio-demographic factors

A total of 424 health professionals were recruited and 406 participants were included in the study with a response rate of 96%. The mean age of respondents with the standard division was 29.6±6.5 years. The age group of the respondents range from 20-65 years and almost half of respondents 209 (51.5%) were in the age range of 20-29 years. Regarding gender 213 (52.5%) were male respondents, and about 225 (55.4%) were single. 206 (50.7%) of the participants were first-degree holders, and 306 (75.4%) of them were with working

experience of less than 7 years. The majority of the respondents 119 (29.3%) were nurses by profession, and 150 (36.9%) of participants' monthly income ranges from 5251-7800 Birr (Table 1).

Table 1: Sociodemo-graphic factors for willingness to use EMR system among health professionals working in Amhara region private hospitals 2021(N=406).

variable	category	Frequency	percent
Sex	male	213	52.5
	female	193	47.5
Age in years	20-29	209	51.5
	30-39	164	40.4
	Above 40	33	8.1
Work experience in year	Less than 7 year	306	75.4
	Above 7 year	100	24.6
religion	Orthodox Christian	315	77.6
	Muslim	71	17.5
	other	20	4.9
Marital status	Single	225	55.4
	Married	166	40.9
	Divorced	11	2.7
	Widowed	4	1.0
Educational level	Diploma	142	35.0
	Degree	206	50.7
	Master and above	58	14.3
Professional category	Physician	38	9.4
	Nurse	119	29.3
	Laboratory	82	20.2
	Pharmacy	71	17.5

	Midwifery<br>Others**	46<br>50	11.3<br>12.3
Monthly income in birr	Less than 3200<br>3201-5250<br>5251-7800<br>7801-10900<br>Above 10901	22<br>145<br>150<br>51<br>38	5.4<br>35.7<br>36.9<br>12.6<br>9.4

** Others include public health, psychiatry, optometry, radiology, physiotherapy, and dental.

Technical Factors for Willingness to Use the EMR System

The majority 312 (76.8%) of health professionals had good knowledge of EMR system use. More than half 257 (63.3%) of the respondents had good skills to use computer systems. Almost half 190 (46.8%) of the respondents did not receive EMR system training because of lack of access for training 105 (55.6%), absence of interest for training 58 (30.5%), lack of time to be self-trained 57 (30%) and 35 (18.4%) of health professionals said as their work was not needed training. About two-thirds, 261 (64.3%) of health professionals had no English language barrier to use a computer and EMR system. 245 (60.3%) of the respondents used a computer device in the EMR system for the following obvious purposes: writing of reports (33.5%), lessening music (16.7%), reading (29.8%), and for keeping patient files (53.9%), (Table 2). The main reasons for not taking EMR training in Amhara region private hospitals was not having access to take EMR system training (Figure 1).

Table 2: technical factors for willingness to use EMR system among health professionals working in Amhara region private hospitals 2021(N=406).

variable	category	frequency	percent

Knowledge on EMR	poor knowledge<br>good knowledge	94<br>312	23.2<br>76.8
Computer skill on EMR	poor skill<br>good skill	149<br>257	36.7<br>63.3
Language difficulty in using EMR	Yes<br>no	145<br>261	35.7<br>64.3
Training on EMR	Yes<br>no	216<br>190	53.2<br>46.8
Reason for not taking EMR training*	No have time to take training<br>No access to take training<br>Not interested to take training<br>My work does not need training	57<br>105<br>58<br>35	30.0<br>55.6<br>30.5<br>18.4
Using a computer for EMR	Yes<br>no	245<br>161	60.3<br>39.7
Reason for using a computer for EMR*	Report writing<br>Keeping patient file<br>Listening music<br>Using internet<br>Reading<br>Others	82<br>41<br>73<br>132<br>77<br>46	33.5<br>16.7<br>29.8<br>53.9<br>31.4<br>18.8

* More than one answer is possible

Figure 1, Reasons for not taking EMR training in Amhara region private hospitals 2021(N=406)

Organizational and Resource-Related Factors

From the total respondents, 230 (56.7%), 225 (55.4%), and 183 (45.1%) were able to access computers, the EMR guideline, and the Internet to run an EMR system, respectively. More than half 212 (52.2%) of healthcare professionals had

been supported by trained IT technical personnel recruited for EMR system maintenance. Nearly half 210 (49.0%) of respondents got managerial support to use the EMR system, however, only 158 (38.9%) of the respondents replayed that adequate budget was allocated for the EMR system, and 168 (41.4%) of respondents were received supportive supervision (M&E) for EMR system (Table 3).

Table 3: technical factors for willingness to use EMR system among health professionals working in Amhara region private hospitals 2021(N=406)

variable	category	frequency	percent
Full computer access (desktop and laptop)	Yes<br>no	230<br>176	56.7<br>43.3
Local area internet access	Yes<br>no	183<br>223	45.1<br>54.9
Having technical support personnel	Yes<br>no	212<br>194	52.2<br>47.8
Having EMR implementation guideline	Yes<br>no	225<br>181	55.4<br>44.6
Having budget allocation for EMR	Yes<br>no	158<br>248	38.9<br>61.1
Having supportive supervision(M&E)	Yes<br>no	168<br>238	41.4<br>58.6
Having management support	Yes<br>no	210<br>196	49.0<br>51.0

Willingness to Use the EMR System

Figure 2, willingness to use EMR system among health professionals working in Amhara region private hospitals 2021(N=406)

Of all study participants, 307 (75.6%), 95% CI (71.4-79.8) had the willingness to use the EMR system in healthcare facilities. About 64.3% and 74.4% of health professionals were willing to purchase and avail personal computer to familiarize with the usage of EMR and undergo a computer training to enable EMR usage, respectively, and also 70.0% were Willingness to use EMR if properly trained (Table 4) and (Figure 2).

Table 4: willingness to use EMR system among health professionals working in Amhara region private hospitals 2021(N=406).

variable	category	frequency	percent
Willingness to undergo computer training to enable usage of EMR	No<br>yes	104<br>302	25.6<br>74.4
Willingness to purchase a personal computer and to familiarize with the usage of EMR	No<br>yes	145<br>261	35.7<br>64.3
Willingness to undergo training on EMR and its implementation	No<br>yes	116<br>290	28.6<br>71.4
Willingness to use EMR if properly trained	No<br>yes	122<br>284	30.0<br>70.0
Willingness to use EMR if the technical infrastructure is available	No<br>yes	127<br>279	31.3<br>68.7
Overall willingness to use EMR system	Not willing<br>willing	99<br>307	24.4<br>75.6

Factors Associated with Willingness to Use the EMR System

Bivariable and multivariable binary logistic regression analyses were done to determine the association between the willingness to use the EMR system and independent variables. Accordingly, those variables which had a p-value of less

than 0.2 in the bivariable regression analysis (Educational level, Knowledge on EMR, Computer skill on EMR, Language difficulty in using EMR, Training on EMR, Using a computer for EMR, Full computer access, Having technical support personnel, Having technical support personnel, Having supportive supervision(M&E), Age group, Having management support and Monthly income) were tested at individual level holding other variables constant against the dependent variable and considered for the multivariable regression analysis. In the final multivariable logistic regression model only variables Knowledge on EMR (AOR=1.85, 95% CI (1.004-3.409)), Training on EMR(AOR=3.29, 95% CI (1.353-8.003)), having technical support personnel (AOR=1.92, 95% CI(1.122-3.305)), having supportive supervision (M&E) (AOR=1.97, 95% CI (1.072-3.628)), and Computer skill on EMR(AOR=1.77, 95% CI (1.002-3.148)) were significant at p-value <0.05 with 95% confidence interval.

As shown in the (table5) healthcare professionals who had good knowledge of EMR system software were 1.85 times more likely to be willing to use the EMR system than those who had poor knowledge of EMR system software (AOR=1.85, 95% CI (1.004-3.409)), study subjects who had computer skill were 1.77 times more likely to have the willingness to use the EMR system compared to those with no have computer skill (AOR=1.77, 95% CI (1.002-3.148)). The study also indicated that health professionals who had got an EMR training course were 3.29 times more likely to have the willingness to use the EMR system compared to those who had not got EMR training (AOR=3.29, 95% CI (1.353-8.003)). health professionals who had got technical support from technical support personnel were 1.92 times more likely to have a willingness to use the EMR system compared to those who had not got technical support (AOR=1.92, 95% CI(1.122-3.305)). Respondents working in the presence of supportive supervision(M&E) were 1.97 times more likely to have willing to use the EMR system than their counterparts (AOR=1.97, 95% CI (1.072-3.628)) (Table 5).

Table 5: factors associated with willingness to use EMR among health professionals working in Amhara region private hospitals 2021(N=406).

variable	category	willingness		Crud OR(95%, CI)	adjusted OR(95%, CI)
		Willing	Not Willing		
		No (%)	No (%)		
Educational level	Diploma<br>Degree<br>Master & above	97(23.9)<br>165(40.6)<br>45(11.1)	45(11.1)<br>41(10.1)<br>13(3.2)	1<br>1.86(1.142-3.053)<br>1.60(0.789-3.270)	1<br>1.81 (0.969-3.391)<br>1.18 (0.395-3.541)
Knowledge on EMR	poor knowledge<br>good knowledge	56(13.8)<br>251(61.8)	38(9.4)<br>61(15.0)	1<br>2.79(1.697-4.594)	1<br>1.85 (1.004-3.409)*
Computer skill on EMR	poor skill<br>good skill	93(22.9)<br>214(52.7)	56(13.8)<br>43(10.6)	1<br>2.99(1.880-4.776)	1<br>1.77 (1.002-3.148)*
Language difficulty on EMR	Yes<br>no	99(24.4)<br>208(51.2)	46(11.3)<br>53(13.1)	1<br>1.82(1.149-2.894)	1<br>1.67 (.990-2.833)
Training on EMR	Yes<br>no	185(45.6)<br>122(30.0)	31(7.6)<br>68(16.7)	3.32(2.053-5.388)<br>1	3.29(1.353-8.003)**<br>1
Using computer for EMR	Yes<br>no	198(48.8)<br>109(26.8)	47(11.6)<br>52(12.8)	2.01(1.271-3.179)<br>1	1.09 (0.602-1.973)<br>1
Full(desktop & laptop) computer access	Yes<br>no	192(47.3)<br>115(28.3)	38(9.4)<br>61(15.0)	2.68(1.681-4.272)<br>1	0.69 (0.280-1.724)<br>1
Having a technical support person	Yes<br>no	176(43.3)<br>131(32.3)	36(8.9)<br>63(15.5)	2.35(1.473-3.754)<br>1	1.92 (1.122-3.305)*<br>1
Having supportive supervision(M&E)	Yes<br>no	140(34.5)<br>167(41.1)	28(6.9)<br>71(17.5)	2.12(1.300-3.476)<br>1	1.97(1.072-3.628)*<br>1
Age group	20-29<br>30-39<br>Above 40	164(40.4)<br>121(29.8)<br>22(5.4)	45(11.1)<br>43(10.6)<br>11(2.7)	1<br>0.77(0.478-1.247)<br>0.54(0.248-1.216)	1<br>0.72 (0.420-1.247)<br>0.95 (0.348-2.609)
Having	Yes	156(38.4)	43(10.6)	1.34(0.853-2.123)	0.79 (0.448-1.402)

management support	no	151(37.2)	56(13.8)	1	1
Monthly income	< 3200	18(4.4)	4(1.0)	0.68(0.162-2.863)	0.34 (0.099-1.216)
	3201-5250	101(24.9)	44(10.8)	0.34(0.127-0.950)	0.31 (0.087-1.141)
	5251-7800	118(29.1)	32(7.9)	0.55(0.202-1.547)	0.22 (0.051-1.009)
	7801-10900	37(9.1)	14(3.4)	0.40(0.130-1.232)	0.55 (0.095-3.214)
	>10901	33(8.1)	5(1.2)	1	1

*=P value less than 0.05 **= P value less than 0.01

Discussion

This institution-based cross-sectional survey was conducted to assess the health professional willingness to use EMR and to identify factors affecting willingness to use EMR in private hospitals of the Amhara region.

The respondents of this study confirmed a good willingness toward the usage of EMR. According to these findings, health professionals who were willing to use the EMR system in private hospitals were 75.6%. This finding is in line with a study carried out in private hospitals of India, where 75% were comfortable working on EMRs (13). However, the result of this study is lower than that of a study conducted in Nigeria (Lagos) (19) and in Kenya (20). The plausible reason for this difference could be lead to the inconsistency of the study subjects being included in the studies. Health professionals included in the current study were considered from all levels of private hospitals in the Amhara region, while respondents in Nigeria were taken only from tertiary levels of hospital settings, and respondents from Kenya were taken from both private and governmental hospitals. Hence, the level of infrastructure, resource allocation, managerial support, and skilled manpower also varied across the type of health facility that makes a difference in utilizing the EMR system.

Another study done in Ethiopian health facilities proved that 85.9% of the experts were willing to use the EMR system (9) when compared to this study our study is slightly lower, the possible reason for this slight difference might be the variability of study sample size, in the current study the size of study subject were less than when compared with the previous study. The other probable reason for this inconsistence could be the difference in the study setting, the setting of the previous study done was from Bahirdar health facilities, those health facilities are selected based on their prior accessibility of EMR, but our study setting was all private hospitals in Amhara region, this could affect the willingness of health professionals, since the availability of technical support and infrastructure, training, health workers technology resistance, and administrative problems varied across this health facility that makes a difference (6, 21).

In this study, health professionals who had good knowledge of the EMR systems were more likely willing to use the EMR system as compared to those with poor knowledge of the EMR system. This may be because health professionals who had good knowledge may tend to accept the advantage of technology and be likely to be willing to use the EMR system. As discussed in other studies (9, 22, 23) health professionals who have good knowledge of the EMR system were more likely willing to use the EMR system than their counterparts or have poor knowledge of the EMR system. This is also a good explanation for the need to create awareness and strengthen continual capacity building among less knowledgeable health professionals, to narrow the knowledge gaps found about the EMR systems so that they will have a good willingness to use EMR and develop their knowledge for better use of the EMR system.

Despite the differences in the study participants, experience and availability of a computer EMR training were significantly associated. Health professionals who had trained EMR system were more likely willing to use the EMR system as compared to those health professionals who had not trained any EMR system before, and this is in line with other findings(9, 22, 24-26) this explained by the evidence that training and education usually change people’s views, willingness, knowledge, and skills of health professionals on EMR systems(19).

Computer skill is the pillar of information communication and EMR utilization in the healthcare system; for this reason, more than half (63.3%) of the respondents in the current study were with good skills in computer application. other studies have indicated that there is a relationship between the level of IT skills and the willingness to use electronic medical records and this study was no exception. Respondents whose computer skill was good were more likely willing to use the EMR system than poor computer skill. This finding was in line with the study findings from Ethiopia (9, 23), Nigeria (19, 27). This is because those health professionals with good computer skills and the availability of computers had a direct influence on health professionals' views on computer-based system use. Availability of adequate computers, other resources, training centers, and support from the different organizations were also the likely explanations for the similarity of being a good skill.

Reliable and timely health information is the foundation of health systems action where information and communication technology initiatives such as EMRs enhance the decision-making process. However, it is sometimes not available when required because of poor supportive supervision (M&E). However, supportive supervision was significantly associated with willingness to use EMR in none of our literature. This study truly explained the fact that supportive supervision was an independent determinant for willingness to use the EMR system. This is because technical support supervision involves identifying or equipping a relatively smaller team of technical staff with the relevant skills-set, the right tools, and resources Thus, for the private as well as public health sector to enhance the effectiveness and efficiency of supportive supervision, the technical support supervision approach should be adopted with a major focus on identifying the skill gaps among the technical staff at all levels, Equipping the technical staff with the relevant skills-set, Motivating and retaining technical staff at all levels of health care and Facilitating the technical teams with the right tools' resources(28). For this reason, about 52.2% of the respondents in the current study were working in the presence of technical support personnel, and respondents working in the presence of technical support personnel were more

likely willing to use EMR systems than their counterparts. This finding is supported by the other study from Ethiopia(29), and Saudi Arabia(30). Probably this may happen as long as individuals got access to technical support their willingness to use a computer would increase because technical support would increase technical skill and this will lead to the willingness to use the EMR system. To alleviate this problem adequate training and technical support should be given to health professionals.

Conclusion

The majority of the healthcare professionals showed a better willingness to use the EMR system. However; lack of computer skills, lack of computer training, lack of technical support personnel, lack of EMR system knowledge, and supportive supervision (M&E) were found as factors to affect the willingness to use EMR systems by health professionals. Therefore, stakeholders should enhance EMR training, focus on continuous follow-up, and offer technical support for health professionals.

Author's contributions

AF substantially contributed to the conception and design, analysis, and interpretation of the study. BT, AM SM, and KS were involved in the analysis, interpretation, and all authors participated in drafting the article or revising it critically for important intellectual content, gave final approval of the version to be published.

Acknowledgment

The authors would like to forward the deepest gratitude to the University of Gondar institute of public health for the approval of ethical clearance and financial support for data collection. Next to this, thanks to all data collectors, supervisors, and respondents.

Abbreviations

AOR, Adjusted Odds Ratio; EMR, Electronic Medical Records; HIS, Health Information Systems; HO, Health office; M&E, Monitoring and Evaluation; SPSS, Statistical Package for Social Science

Reference

1. Cesnik B, Kidd MR. History of health informatics: a global perspective. Stud Health Technol Inform. 2010;151:3-8.
2. Al AJ, El-Masri S. Proposal of an architecture for the national integration of Electronic Health Records: a semi-centralized approach. Studies in health technology and informatics. 2012;180:917-21.
3. Walsham G. Health information systems in developing countries: some reflections on information for action. Information Technology for Development. 2020;26(1):194-200.
4. Bazile EP. Electronic medical records (EMR): An empirical testing of factors contributing to healthcare professionals' resistance to use EMR systems. 2016.
5. Hamilton C. The WHO-ITU national eHealth strategy toolkit is an effective approach to national strategy development and implementation. Studies in health technology and informatics. 2013;192:913.
6. Chebole GC. Factors influencing adoption of electronic medical record systems in public health facilities in Kenya: a case of Nakuru county. 2015.
7. Ahmad BIe. User acceptance of health information technology (HIT) in developing countries: a conceptual model. Procedia Technology. 2014;16:1287-96.
8. Paré G, Raymond L, de Guinea AO, Boba-Nzaou P, Trudel M-C, Marsan J, et al. Electronic health record usage behaviors in primary care medical practices: A survey of family physicians in Canada. International Journal of Medical Informatics. 2015;84(10):857-67.
9. Berihun B, Atnafu DD, Sitotaw G. Willingness to Use Electronic Medical Record (EMR) System in Healthcare Facilities of Bahir Dar City, Northwest Ethiopia. BioMed Research International. 2020;2020.
10. Ethiopia moh. Health Sector Transformation Plan II - Family Planning 2020.
11. Asubonteng K. THE POTENTIAL FOR PUBLIC-PRIVATE PARTNERSHIP (PPP) IN ETHIOPIA. Ethiopian chamber com. 2011.
12. O'Mahony D, Wright G, Yogeswaran P, Govere F. Knowledge and attitudes of nurses in community health centers about electronic medical records. curationis. 2014;37(1):01-6.

13. Pera NK, Kaur A, Rao R. Perception of electronic medical records (EMRs) by nursing staff in a teaching hospital in India. International Journal of Advanced Medical and Health Research. 2014;1(2):75.
14. Ahmed MH, Bogale AD, Tilahun B, Kalayou MH, Klein J, Mengiste SA, et al. Intention to use electronic medical record and its predictors among health care providers at referral hospitals, north-West Ethiopia, 2019: using unified theory of acceptance and use technology 2 (UTAUT2) model. BMC Medical Informatics and Decision Making. 2020;20(1):1-11.
15. Msukwa MK. User perceptions on electronic medical record system (EMR) in Malawi. Published thesis, University of Malawi. 2011.
16. Organization WH. Medical records manual: a guide for developing countries: Manila: WHO Regional Office for the Western Pacific; 2006.
17. Yeshimebet K. Revitalizing The Smartcare System Of Surgery Inpatient Department In Saint Paul Hospital Millennium Medical College. 2014.
18. Mekonnen ZA, Gelaye KA, Were MC, Tilahun BJBH, Informatics C. Mothers intention and preference to use mobile phone text message reminders for child vaccination in Northwest Ethiopia. 2021;28(1).
19. Onigbogi O. Doctors' Attitude and Willingness to Use Electronic Medical Records at the Lagos University Teaching Hospital, Lagos, Nigeria. Online Journal of Public Health Informatics. 2018;10(2).
20. Mercy Gacheri Nkanata EOM, Grace Irura. Comparative Analysis of Hospital Information Management Systems Among Healthcare Workers in Two Selected Hospitals in Kenya. Library Philosophy and Practice(e-journal). 2018;2056.
21. Spaulding RJ, Russo T, Cook DJ, Doolittle GCJJot, telecare. Diffusion theory and telemedicine adoption by Kansas health-care providers: Critical factors in telemedicine adoption for improved patient access. 2005;11(1_suppl):107-9.
22. Oumer A. Utilization of Electronic Medical Record and Associated Factors Among Health Professionals in Public Health Facilities With Service Delivery, Eastern Ethiopia. 2020.
23. Biruk S, Yilma T, Andualem M, Tilahun BJBmi, making d. Health Professionals' readiness to implement electronic medical record system at three hospitals in Ethiopia: a cross-sectional study. 2014;14(1):1-8.
24. Yehualashet G, Asemahagn M, Tilahun B. The attitude towards and use of electronic medical record system by health professionals at a referral hospital in northern Ethiopia: Cross-sectional study. Journal of Health Informatics in Africa. 2015;3(1).
25. Tilahun B, Fritz F. Comprehensive evaluation of electronic medical record system use and user satisfaction at five low-resource setting hospitals in Ethiopia. JMIR medical informatics. 2015;3(2):e22.

26. Awol SM, Birhanu AY, Mekonnen ZA, Gashu KD, Shiferaw AM, Endehabtu BF, et al. Health professionals’ readiness and its associated factors to implement electronic medical record system in four selected primary hospitals in Ethiopia. 2020;11:147.
27. Poluyi A, Onigbogi O, Poluyi E. An assessment of the knowledge of medical doctors on Electronic Medical Records in a Tertiary Health Center in Sub-Saharan Africa: A cross-sectional study. 2019.
28. health Mo. national support supervision guidelines for health service. the republican of Uganda. 2020.
29. Yesef G/Egziheber DRB, Addise DM. challenges associated with physician acceptance and usage of electronic medical records in public hospitals of Addis Ababa. 2013.
30. Aldosari B, Al-Mansour S, Aldosari H, Alanazi AJIiMU. Assessment of factors influencing nurses' acceptance of electronic medical records in a Saudi Arabia hospital. 2018;10:82-8.

figure 2

Click here to access/download;Figure;Figure 2.docx

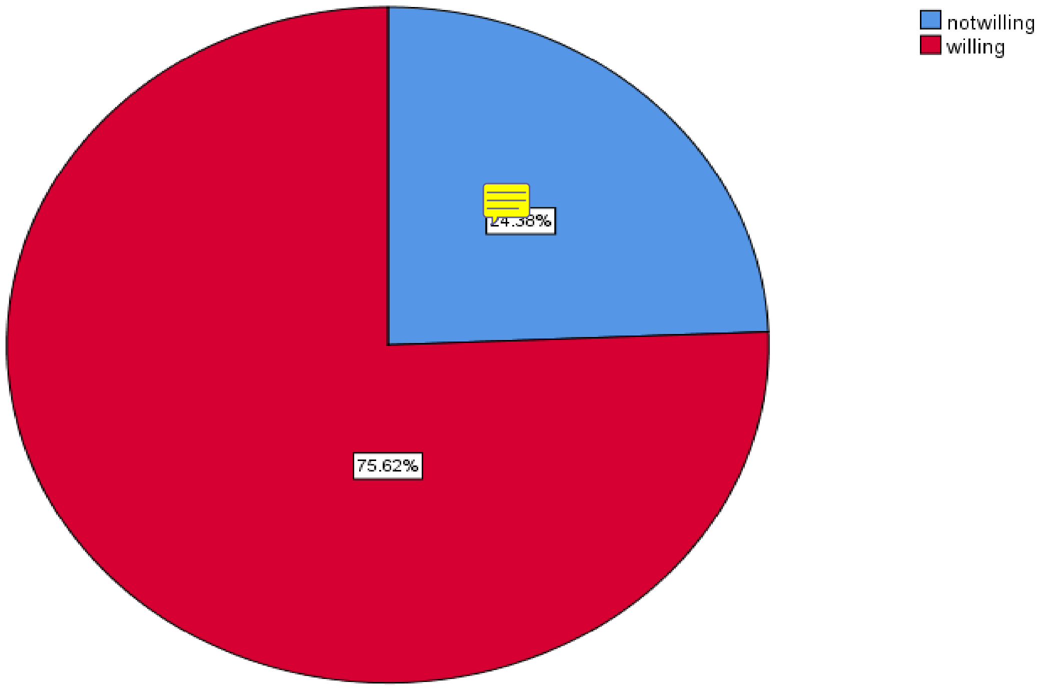

figure 1 Click here to access/download;Figure;Figure 1.docx

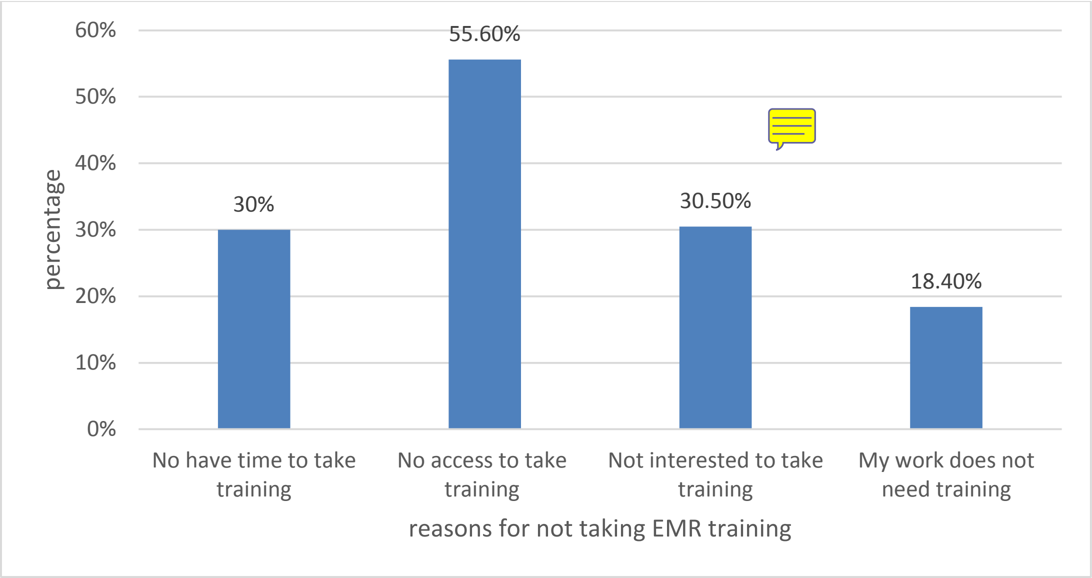

Supplement: Attachment — Submitted filename: PONE-D-22-11490 (1) (1) CORRECTED.pdf [file pone.0282044.s004.pdf]
