# Supplementary material for: Willingness to use electronic medical record (EMR) system and its associated factors among health professionals working in Amhara region Private Hospitals 2021, Ethiopia
Source: PLoS One. 2023 May 1;18(5):e0282044. doi: 10.1371/journal.pone.0282044 (PMC10150984; doi:10.1371/journal.pone.0282044)
Supplement: Attachment — Submitted filename: Plos one -Review.docx [file pone.0282044.s003.docx]

**Abstract**

1. Never use the abbreviation on the abstract…
2. How was this statement correct” Descriptive statistics with Binary logistic regression was performed to estimate the crude and adjusted odds ratio with a 95% Confidence interval? How descriptive statistics was regressed for logistic regression.
3. Do not begin with number
4. The conclusion is not SMART which is a carbon copy of the results without any change. In my view better to re-construct the conclusion in a manner that addresses the research question.
5. At conclusion, Why to say high, what is your ground to say as such?

**Background**

6. There are a # of editorial issues which needs English language subject expert editing
7. It is too long please shorten it focusing on :-
   i. What is known on HIT & EMR?
   ii. What is unknown about HIT & EMR, that you want to address by conducting this study?
   iii. What are the major gaps in EMR in Ethiopia particularly in the private sector?
8. I have-not seen anything about magnitude & coverage of EMR in private sectors & what about the important factors influencing it?
9. What is the status of EMR implementation in private sectors...?

**Methods**

10. Why took 50% of willing to use EMR as a proportion; why not search for actual proportions, why not use my articles??? As a result I did not believe that it was representative. What bout for the second objective…?
11. Do you think that 13 Question is enough to assess level of knowledge...?
12. What about the cluster variability, do you know the facilities in Amhara region is with homogeneous characteristics...?
13. What makes this study different from studies conducted by myself and Dr. Mulusew Andualem’s article..? You need to justify how it differed from?
14. What does it mean by technical factor mean...? I think needs to be operationalized. Then; I think to technique mean sth related to skill. If so how can we related & categorize for knowledge technical factors... despite it might a prerequisite for skill that someone will develop.
15. I suspect a kind of plagiarism & what is important to repeat the same study within two years in the same population/area, I think wasting of resources.
