# Supplementary material for: Willingness to use electronic medical record (EMR) system and its associated factors among health professionals working in Amhara region Private Hospitals 2021, Ethiopia
Source: PLoS One. 2023 May 1;18(5):e0282044. doi: 10.1371/journal.pone.0282044 (PMC10150984; doi:10.1371/journal.pone.0282044)
Supplement: Attachment — Submitted filename: Response to Reviewers.docx [file pone.0282044.s005.docx]

**Point -by- point response**

**Subject: Revision and resubmission of the manuscripts** PONE-D-22-11490

**To:  To Editor of plos one Journal.**

We want to convey our appreciation for reviewing our research to resubmit the revised manuscript, titled ”Willingness to Use Electronic Medical Record (EMR) System and its Associated Factors among Health Professionals Working in Amhara region Private Hospitals 2021, Ethiopia**."**.

We appreciate the time and efforts of the editor and the reviewers who have dedicated themselves to providing your valuable feedback on our manuscript. We are grateful for your insightful comments on our paper. We have been able to incorporate changes to almost all of the suggestions provided by the reviewers. We highlighted (yellow) text the changes within the revised manuscript.

**With kind regards!**

On behalf of the co-authors

Andualem Fentahun (MPH in Public Health Informatics)

Corresponding author

**Response to reviewers’ comments**

We want to thank the reviewers for their insightful comments, which helped improve the paper's overall quality and readability. The comments, suggestions, and feedback were positive, and most of the reviewers’ comments and concerns were followed. The highlighted (yellow color) file highlights specific document changes in the revised manuscript. Please see the blue text below for a detailed response to the reviewers' complaints and suggestions.

Here is a point-by-point response to the reviewers’ comments and concerns.

**Comments from reviewer 1**

Comment 1: Never use the abbreviation on the abstract…

Response: Dear reviewers, Thank you for pointing out such important suggestions and valid comments, based on your comments we amend and exclude abbreviations in abstract part of revised manuscripts like EMR=electronic medical record, but other abbreviations like CI, AOR and SPSS are common and known abbreviations.

Comment 2: How was this statement correct” Descriptive statistics with Binary logistic regression was performed to estimate the crude and adjusted odds ratio with a 95% Confidence interval? How descriptive statistics was regressed for logistic regression.

Response: dear reviewers, thank you for reminding us to revise this section. We made the revision on abstract part of revised manuscript based on your concern, and we changed “descriptive statistics with logistic regression” in to “descriptive statistics and logistic regression” to make it clear from abstract part in method section of revised manuscript line number 5.

Comment 3: Do not begin with number

Response: dear reviewers, thank you for reminding us to revise this section, we made the revision on abstract part of revised manuscript and highlighted the change.

Comment 4: The conclusion is not SMART, which is a carbon copy of the results without any change. In my view better to re-construct the conclusion in a manner, that addresses the research question.

Response: Dear Reviewers, We completely accepted your suggestion and comments; we revised and reconstructed the smart conclusion based on your concerns by drawing conclusion appropriately based on the data presented and based on its objective.

Comment 5: At conclusion, Why to say high, what is your ground to say as such?

Response: dear reviewers, Thank you for raising such important question, our ground to say high proportion was the finding result (75.6), and the result is above the half, but now we modified the term high in to good and incorporated it in the abstract part of conclusion section of revised manuscript.

Comment 6: There are a # of editorial issues, which needs English language subject expert editing

Response: dear reviewers, Thank you for the great suggestion. We completely accepted your suggestion, and we revised entire manuscript editorial issues and English language edition with experts as much as possible to increase the readability of our manuscript.

Comment 7: It is too long please shorten it focusing on:-

Response: dear reviewer, Thank you for this suggestion, based on your comment (i,ii,iii) we have revised the manuscript by shortening, and your suggestion is included under the introduction section of revised manuscript.

Comment 7.i: What is known on HIT & EMR?

Response: dear reviewers, Thank you for suggesting this important suggestions, we incorporated your suggestion by clearly stating what is known in EMR and HIT (i.e. in our sense HIT means HIS) in the introduction section Line number 3-5, 28-34, and 52-55.

Comment 2.ii: What is unknown about HIT & EMR, that you want to address by conducting this study?

Response: dear revisers, Thank you again for this suggestion, based on your comment we have revised the manuscript by clearly stating what is unknown on EMR, and your suggestion is included under the introduction section line number 20-25 and 36-44.

Comment 7.iii: What are the major gaps in EMR in Ethiopia particularly in the private sector?

Response: dear reviewers, we completely accepted your suggestion. We revised and added major gaps of EMR in private sectors to the revised manuscript by including your suggestions under the introduction section line number 30-35

Comment 8: I have-not seen anything about magnitude & coverage of EMR in private sectors & what about the important factors influencing it?

Response: dear reviewers, Thank you for giving your time to review our paper. Even though the magnitude and coverage of EMR in private sector is not widely available in published literatures, we tried to include your comment by founding different data on the influencing factors and coverage of EMR in private sectors under the introduction section line number 11-18.

Comment 9: What is the status of EMR implementation in private sectors...?

Response: dear reviewers: Thank you for raising such important question. Of course, we stated the status of EMR implementation in private sector a little, even though the status EMR implementation in Ethiopian private sector is not known in scientific way, we got the status through asking the private hospitals and we included it in introduction section of revised manuscript line number 12-15.

Comment 10: Why took 50% of willing to use EMR as a proportion; why not search for actual proportions, why not use my articles??? As a result, I did not believe that it was representative. What bout for the second objective…?

Response: dear reviewers, Thank you so much for valuable comments what you raised on taking 50% proportion, we have used 50% proportion for willingness to use EMR in private hospital because there is no study conducted related with willingness to use EMR in Ethiopia. Sorry, we are not able to distinguish which one your article is? If it is mr, Birhanu`s Article conducted in Bahirdar city, this article is not conducted necessarily on private hospital rather it is conducted in Dahrrdar public health facilities.

For second objective, there is no other study conducted in Ethiopia related with second objective in private hospitals.

Comment 11: Do you think that 13 Question is enough to assess level of knowledge...?

Response: dear reviewers, thank you for raising the question unclear with you. Yes, 13 question is enough to assess level of knowledge because these questions was taken from other study and adapted in to our study by pre testing and validating with experts revision before data collection, and then by categorizing Likert scale value in to good knowledge and poor knowledge. Beside that reliability test were performed.

Comment 12: What about the cluster variability, do you know the facilities in Amhara region is with homogeneous characteristics...?

Response: dear reviewers, thank you for your important and constructive comment. Of course you may think the cluster variability in different facilities, But the study setting was one region, the characteristics of hospitals in one region has homogenous characteristics, since the level of facility is the same hospitals, with the same geographical area in Ethiopia. If it was different level of health facility like health center, clinics and hospitals, it may happen.

Commnet 13: What makes this study different from studies conducted by myself and Dr. Mulusew Andualem’s article..? You need to justify how it differed from?

Response: dear reviewers, thank you for you important questions. The study conducted by Dr. mulusew Andualem, and you is different from me by:

In terms of ownership: your study is public or not all health facility you studied owned by private, whereas this study is only private owned or private hospitals.

In terms of facility: your study is in different health facility including clinic, health centers and hospitals, whereas this study is conducted in hospitals only

In terms of setting: your study is only in bahirdar city, where as this study is including all cities in Amhara region having private hospitals (4 city)

In terms of sample size, sampling technique and source population, and this article is new and original since there is no study conducted in private hospitals in Ethiopia about willingness to use EMR.

Comment 14: What does it mean by technical factor mean...? I think needs to be operationalized. Then; I think to technique mean sth related to skill. If so how can we related & categorize for knowledge technical factors... despite it might a prerequisite for skill that someone will develop.

Response: dear reviewers, Thank you for raising such important question and also thanks for reminding us to operationalize the term technical factors, dear reviewer based on your suggestion we operationalized technical factors, but when we say technical factor, it is general term, which can incorporate computer related terms in addition to skills under the method section.

Dear reviewer, thank you very much for giving comments on how can to categorize knowledge on technical factors without operationalizing it, even though during proposal development from conceptual diagram we categorize knowledge under technical factor without operationalizing, now we operationalize technical factors as it includes knowledge in addition to other technical related factors.

Comment 15: I suspect a kind of plagiarism & what is important to repeat the same study within two years in the same population/area, I think wasting of resources.

Response: dear reviewers, Thank you for raising your idea on plagiarism and on being the same study. However, our study is original and novel on private sector, since private sectors health structure is different from government owned. Even though the population is health professional, they are in private hospitals and study area is different from your studies. For your suspecting of plagiarism, we never take direct copy of another idea, rather we support another idea by citing properly.

**Comments from reviewer 2**

Reviewer #2: “This Topic will attract wide readership. Its original and contributes to knowledge.”

Response: dear reviewers, Thank you very much for your appreciation and your recognition of our study.

**Overall conclusion**

We would like to thank the editor, and the reviewers for your excellent and genuinely positive reviews of our manuscript. We agree with and accept all comments and suggestions in the manuscript. We hope we read and looked at all the comments carefully, amended them accordingly, and highlighted them in the revised manuscript. We take editors comment and incorporate broad and specific objective, exclude abbreviations and authors contribution from document, and also we exclude funding related idea from acknowledgement part.

We believe and hope this study will contribute to planning and decision making of governments and private hospital owners as it is the first study in Ethiopia. We look forward to hearing from you regarding our submission and responding to any further questions or comments you may have.

*Sincerely,*

*Andualem Fentahun Senishaw*
