# Supplementary material for: Willingness to use electronic medical record (EMR) system and its associated factors among health professionals working in Amhara region Private Hospitals 2021, Ethiopia
Source: PLoS One. 2023 May 1;18(5):e0282044. doi: 10.1371/journal.pone.0282044 (PMC10150984; doi:10.1371/journal.pone.0282044)
Supplement: Attachment — Submitted filename: Response to Reviewers.docx [file pone.0282044.s006.docx]

**Point -by- point response**

**Subject: Revision and resubmission of the manuscripts** PONE-D-22-11490

**To:  To Editor of plos one Journal.**

We want to convey our appreciation for reviewing our research to resubmit the revised manuscript, titled” Willingness to Use Electronic Medical Record (EMR) System and its Associated Factors among Health Professionals Working in Amhara region Private Hospitals 2021, Ethiopia**."**.

We appreciate the time and efforts of the editor and the reviewers who have dedicated themselves to providing your valuable feedback on our manuscript. We are grateful for your insightful comments on our paper and we send the paper for professional proofreading service to improve English language editorial issues. We have been able to incorporate changes to almost all of the suggestions provided by the reviewers. We highlighted (yellow) text the changes within the revised manuscript.

**With kind regards!**

On behalf of the co-authors

Andualem Fentahun (MPH in Public Health Informatics)

Corresponding author

**Comments from reviewer 1**

Comment 1: Still the conclusion needs to be revised in a manner that the take of massage is boldly communicated

Response: Dear reviewers, thank you for giving chance to revise again in conclusion part, based on your comments we amend the conclusion in a manner leads to communicate message boldly.

Comment 2: The queries related the issue of sampling is not yet addressed.

Response: dear reviewers, thank you for your comment on sampling issues, we determined the sample size of this study by using a single population proportion formula: Since we cannot find any study conducted in Ethiopia to assess the willingness to use the EMR systems and associated factors among health professionals working in private hospitals. Therefore, by assuming a proportion (p) of 50% of willing to use the EMR system and its associated factors finally we got 423 final sample size

There were 10 private hospitals in Amhara region, from these 10 hospitals, health professionals working permanently were included for study, and then we proportionally allocated for each hospital based on actual number, since health professionals working in these 10 private hospitals were homogenous. Finally, we use simple random sampling method to get final sample.

Comment3: The issues of plagiarism is not narrating in away that is convincing

Response: dear reviewers, thank you very much for giving comment to convince plagiarism issues. In the first review the concern of plagiarism and similarity of our papers with other study was raised. Now we fixed the problem of plagiarism by detecting line by line and by using plagiarism detector software and also we paraphrased by using different tools. We provide more information about the similarity or plagiarism of our paper with other study by clarifying and adding further convincing ideas in addition to the previous response, so this paper is different from the other studies: first we highly appreciate and acknowledge your work to do and publish your novel ideas through scientific way. Because we used your study as a reference by acknowledging or citing properly when there was the need of supporting our study by other scientific articles. But we are not plagiarized/copy completely as our work rather we support our idea in context which need to be supported by your study. Our intention of doing this research is to know the willingness of health professionals to use the EMR system in private hospitals not in all private health facilities, and not only in one city rather in entire regions of Amhara private hospitals, so this is the first study conducted in private hospitals to assess the willingness to use EMR system. This can be an input for private hospitals to plan and manage their future interest to implement and use the EMR system.

Comment 4: there is problem of English language editorials

Response: thank you very much dear reviewers, we accepted your comment without any doubt, now we try to fix English language editing problem by sending to experts for further proofing and also, we used different grammar and English language editor tools to correct it. So now we believe corrected.

Comment 5: Questions # 12 & 14 has to be seen carefully & addressed

Response: dear reviewers, thank you to give a chance to address further carefully.

aPrevious Question # 12 says What about the cluster variability, do you know the facilities in Amhara region is with homogeneous characteristics...?

response: dear reviewer we incorporate another reasons in addition to previous response to make understandable and logical, we think cluster variability may not be an issue in this paper because, the characteristics of hospitals in one region has homogenous characteristics, with the same geographical area. Even if we think cluster variability, clusters or facilities should be heterogenous and more than 20. But our facilities or hospitals are homogenous and few in number of clusters. Cluster variability increases as the number of cluster increases. to say cluster variability happen it should be above 30 and 50 clusters.

Previous question # 14: What does it mean by technical factor mean...? I think needs to be operationalized. Then; I think to technique mean sth related to skill. If so how can we related & categorize for knowledge technical factors... despite it might a prerequisite for skill that someone will develop.

Response: dear reviewer we acknowledge your comment but we operationalize technical factor in our context to incorporate knowledge in addition to other technical factor like this,” Technical factors: - which are technical and knowledge-related factors to use EMR in private hospitals, such as computer skills, knowledge of EMR, language difficulty, training, and using a computer for EMR”. Just we operationalize technical factor to incorporate knowledge and to make paper comprehensive in layout out each section.

**Finally,** we would like to thank the editor, and the reviewers for your excellent and genuinely positive reviews of our manuscript. We agree with and accept all comments and suggestions in the manuscript. We hope we read and looked at all the comments carefully, amended them accordingly, and highlighted them in the revised manuscript. We take editors comment and we tried to cover all your comments

We believe and hope this study will contribute to planning and decision making of governments and private hospital owners as it is the first study in Ethiopia.

*Sincerely,*

*Andualem Fentahun Senishaw*
